# Assessing size of pituitary adenomas: a comparison of qualitative and quantitative methods on MR

**DOI:** 10.1007/s00701-015-2699-7

**Published:** 2016-01-29

**Authors:** Benjamin M. Davies, Elizabeth Carr, Calvin Soh, Kanna K. Gnanalingham

**Affiliations:** 1 Department of Neurosurgery, Greater Manchester Neurosciences Centre, Salford Royal Foundation Trust, Stott Lane, Salford, M6 8HD Manchester UK; 2 Department of Neuroradiology, Greater Manchester Neurosciences Centre, Salford Royal Foundation Trust, Stott Lane, Salford, M6 8HD Manchester UK; 3 Manchester Academic Health Sciences Centre, University of Manchester, Manchester, UK

**Keywords:** Pituitary tumour, Hardy grade, Knosp grade, Ellipsoid volume, Perimeter volume, Planimetry, Volumetric analysis, Magnetic resonance imaging

## Abstract

**Background:**

A variety of methods are used for estimating pituitary tumour size in clinical practice and in research. Quantitative methods, such as maximum tumour dimension, and qualitative methods, such as Hardy and Knosp grades, are well established but do not give an accurate assessment of the tumour volume. We therefore sought to compare existing measures of pituitary tumours with more quantitative methods of tumour volume estimation.

**Method:**

Magnetic resonance imaging was reviewed for 99 consecutive patients with pituitary adenomas awaiting surgery between 2010 and 2013. Maximal tumour diameter, Hardy and Knosp grades were compared with tumour volume estimates by the ellipsoid equation, [$\frac{4}{3}\pi\ (a.b.c)$], (i.e. ellipsoid volume) and slice-by-slice perimetry (i.e. perimeter volume).

**Results:**

Ellipsoid and perimeter methods of tumour volume estimation strongly correlated (*R* ^2^ = 0.99, *p* < 0.0001). However the correlation was less strong with increasing tumour size, with the ellipsoid method slightly underestimating. The mean differences were −0.11 (95 % CI, −0.35, 0.14), −0.74 (95 % CI, −2.2, 0.74) and −1.4 (95 % CI, −6.4, 3.7) for micro-tumours, macro-tumours and giant tumours respectively. Tumour volume correlated with maximal diameter, following a cubic distribution. Correlations of tumour volume with Hardy and Knosp grades was less strong.

**Conclusions:**

Perimeter and ellipsoid methods give a good estimation of tumour volume, whereas Knosp and Hardy grades may offer other clinically relevant information, such as cavernous sinus invasion or chiasmal compression. Thus the different methods of estimating tumour size are likely to have different clinical utilities.

## Introduction

Pituitary tumours are common intracranial lesions, with an estimated prevalence of 10–17 % [3, 4]. Pituitary adenomas are the commonest type of pituitary tumour and can present in a variety of shapes and sizes. Most lesions are small and inconsequential, but a proportion manifest with clinical symptoms and require intervention [14]. Symptoms usually develop through changes in pituitary hormone function, compression of the optic chiasm or invasion into the cavernous sinus. Magnetic resonance (MR) imaging is the mainstay of diagnosis and surveillance, including assessment of response to treatment.

Various methods for reporting pituitary adenoma size and shape exist. Current convention across oncology [RECIST 1.1] recommends that a maximal dimension is adequate for the reporting of tumour size, and this is also commonplace in the clinical management of pituitary tumours [2]. However, such simplicity comes with an accepted inaccuracy as changes in tumour diameter have a cuboidal relationship with changes in tumour volume [1]. Categorical systems, such as those described by Hardy and Knosp, circumvent some of these limitations by representing common patterns of pituitary tumour growth and qualitatively indicating size. However, these are rarely used clinically and instead are more common in the research setting [5, 11].

Assessment of pituitary tumour volume is an attractive and likely more accurate alternative [1]. Options of assessing pituitary tumour volume include estimation based on geometric models, such as the ellipsoid equation [$\frac{4}{3}\pi\ (a.b.c)$] or three-dimensional (3D) segmentation using slice-by-slice perimetry [7, 13].

Our aim was to compare these methods of tumour volume estimation in pituitary tumours and to consider their relationship with the existing descriptive measures; namely, maximal tumour diameter, Hardy grade and Knosp grade. To our knowledge, this has not previously been considered.

## Methods

Consecutive patients undergoing endoscopic trans-sphenoidal surgery for a pituitary adenoma under a single neurosurgeon, between 2010 and 2013 were identified from the departmental database, compiled prospectively. Patient sex, age and tumour histology were noted. Given the potential difficulty in identifying residual tumour from postoperative changes on MR, patients undergoing revision surgery were excluded in this initial study.

**Fig. 1:**
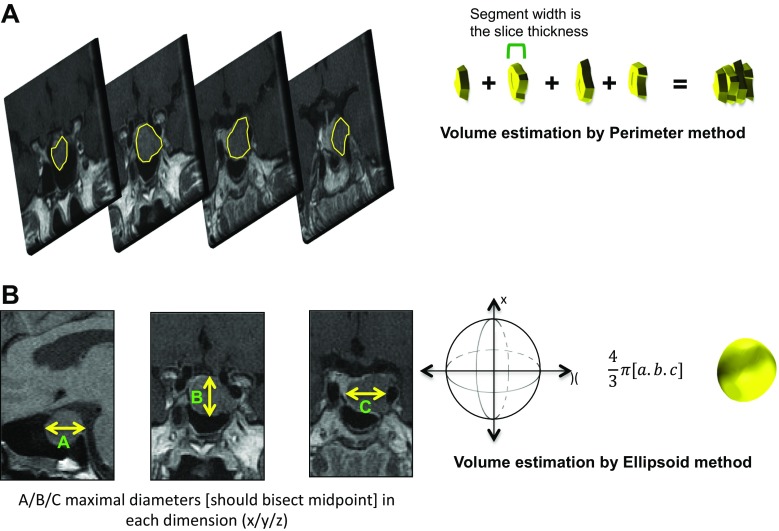
Pituitary tumour volume estimation. **a** The perimeter method requires the tumour to be outlined manually and by taking the thickness of imaging slice, a volume can be calculated [15]. The volume is then added together for each slice. **b** The ellipsoid method requires maximal diameters to be recorded in each dimension and fed into the ellipsoid equation [15]

**Fig. 2:**
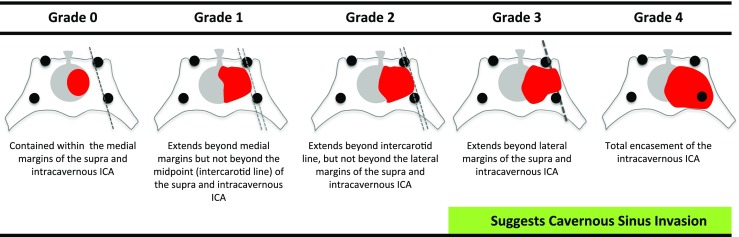
Knosp grade. This system grades the parasellar extension of the tumour towards the cavernous sinus in relation to the intracavernous carotid artery (ICA) [11]

**Fig. 3:**
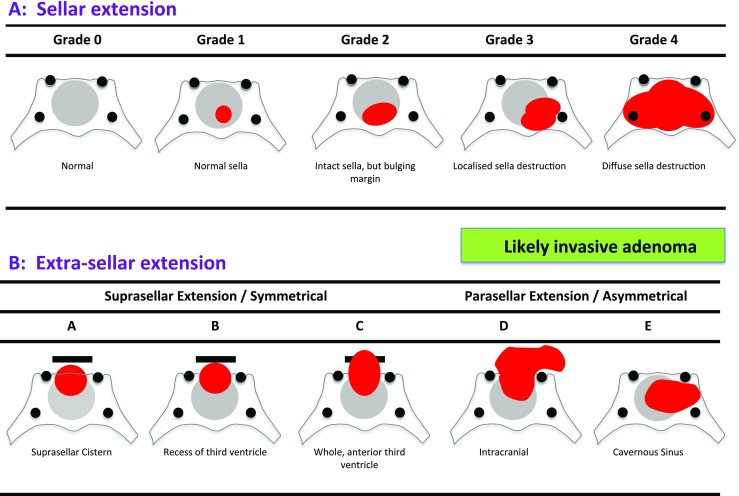
Hardy grade. This system considers both the sella disruption (**a**), denoted by numbers *0–4*, and the type of suprasellar extension (**b**) denoted by letters *A–E* [5]

Preoperative MR imaging, undertaken on a 1.5-T scanner, was reviewed using a Centricity PACS workstation (GE Healthcare, Chalfont St Giles, UK) to ascertain the tumour’s maximal diameter, ellipsoid and perimeter volumes, Hardy and Knosp grades (Figs. 1, 2 and 3) [5, 11]. Perimeter volume was calculated by manual slice-by-slice segmentation, also known as planimetry (tracing the tumour outline), on coronal views and allowing the Centricity workstation to create a 3D reconstruction and volume (Fig. 1a). Ellipsoid volume was calculated using the formula $\frac{4}{3}\pi \left[a.b.c\right]$, where *a*, *b* and *c* are the maximal orthogonal diameters in each dimension (Fig. 1b).

Tumours were subcategorised as micro-tumour (diameter <1 cm; volume <0.52 cm^3^), macro-tumour (diameter 1–2.5 cm; volume 0.52–8.16 cm^3^) or giant tumour (diameter >2.5 cm; volume >8.16 cm^3^) in size.

The Knosp grade describes tumour encroachment or invasion into the cavernous sinus (Fig. 2) [11]. The Hardy grade is composed of two separate ordinal classifications, one denoted by numbers describing the sella disruption and the other by letters describing the suprasellar extension, and for the purpose of correlations, these were considered separately (Fig. 3) [5].

A randomly selected sub-set of 31 patients (17 men, 14 women; mean age of 53 ± 16 years) were also graded by a second observer, to assess inter-observer variability.

### Statistical analysis

Statistical analysis was performed using SPSS version 20.0 (Chicago, IL). Significance was set at *p* < 0.05. Tumour grades were compared as a single group and in their separate subcategories. Pearson’s correlation coefficient was used to study correlations between continuous variables and Spearman’s *rho* for ordinal variables. The Bland-Altman method was used to assess agreement, firstly between the perimeter and ellipsoid estimates of volume and secondly between the two observers.

## Results

In total, 99 patients (54 male, 45 female) with a mean age of 55 ± 15 years were included. The majority were non-functioning pituitary adenomas [NFPAs, *n* = 57]. Functioning tumours included acromegaly (*n* = 21) and Cushing’s disease (*n* = 11). The remaining pathology were pituitary apoplexy (*n* = 4), prolactinomas (*n* = 3) or cystic pituitary lesions (*n* = 3). These tumours had a wide range of tumour size and grades (Table 1). NFPAs, on average, had a larger size and grades than functioning pituitary tumours.

**Table 1:** Pituitary tumour characteristics by pathology type. Average volume and maximal diameter are represented as means (± SD), whilst average grades of tumour as the median values

Pathology	*n*	Perimeter volume (cm^3^)	Ellipsoid volume (cm^3^)	Maximal diameter (cm)	Hardy number (median)	Hardy letter (median)	Knosp (median)
NFPA	57	8.6 ± 10	7.4 ± 9	27.2 ± 11	2	C	1
Acromegaly	21	4.7 ± 9	4.2 ± 8	19.5 ± 11	2	A	0
Cushing’s Disease	11	2.0 ± 6	2.4 ± 7	10.9 ± 10	1	A	0
Other	10	4.9 ± 6	4.4 ± 6	22.2 ± 11	2	A	2
	99	6.7 ± 9	5.9 ± 8	23.2 ± 12	2	C	0

**Fig. 4:**
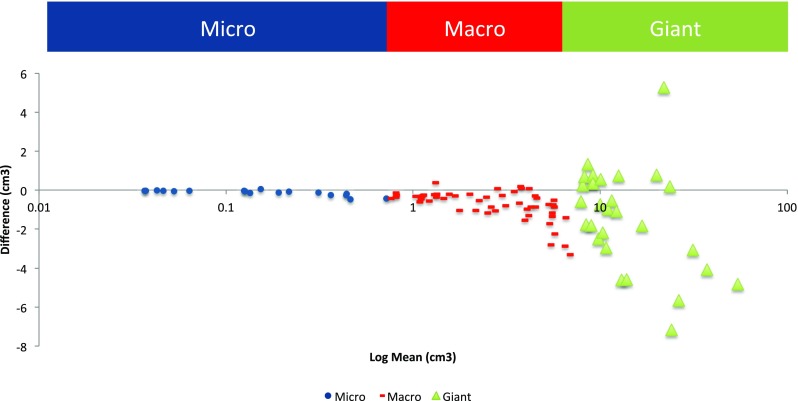
Bland-Altman comparison of perimeter and ellipsoid methods of tumour volume estimation. Micro-tumours (diameter <1 cm; <0.52 cm^3^, *blue dots*), macro-tumours (diameter 1–2.5 cm; 0.52–8.16 cm^3^, *red lines*) and giant tumours (diameter >2.5 cm; >8.16 cm^3^, *green triangles*) are distinguished separately. Agreement between the two methods is less close for the giant adenomas

Estimations of tumour volume by ellipsoid and perimeter methods strongly correlated (*R* ^2^ = 0.99, *p* < 0.0001). Bland-Altman analysis found their mean difference overall to be −0.82 (95 % CI, −3.8, 2.2), although the analysis also demonstrated that the magnitude of difference increased with tumour size; −0.11 (95 % CI, −0.35, 0.14), −0.74 (95 % CI, −2.2, 0.74) and −1.4 (95 % CI, −6.4, 3.7) for micro-tumours, macro-tumours and giant tumours respectively (Fig. 4). When this difference was represented as a proportion of average measured tumour volume, this percentage error decreased to 59 %, 24 % and 9 %, respectively.

**Fig. 5:**
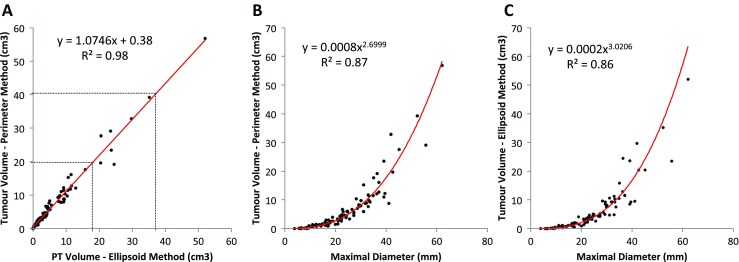
Scatter plots describing the relationship between perimeter and ellipsoid methods of estimating tumour volume (**a**) and their relationship with maximal diameter (**b** and **c** respectively). Lines of best fit have been plotted

In the majority of cases (*n* = 81, 82 %) the perimeter method calculated a slightly larger volume than the ellipsoid method (Fig. 5a). When perimeter volume was plotted against ellipsoid volume, a *y* intercept of 0.35 cm^3^ was calculated, indicating that when an ellipsoid volume of near 0 cm^3^ is calculated, the perimeter method would still find a substantial volume.

Inter-observer error for estimation of the ellipsoid volume (mean difference, 0.4 cm^3^; 95 % CI, 0.3, 1.2) was less than for the perimeter volume (mean difference, 0.7 cm^3^; 95 % CI, 0.2, 1.7), with coefficients of variation of 8.2 % compared with 11.3 %.

**Fig. 6:**
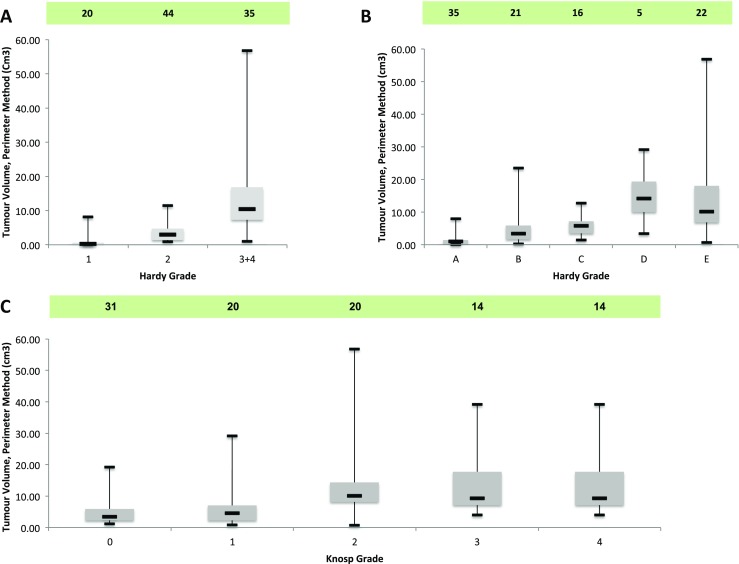
Box-plots denoting the relationship between Hardy numbers (**a**) [sella disruption] and Hardy letters (**b**) [suprasellar extension] and Knosp grade (**c**), with tumour volume estimated by the perimeter method. The box plots depict the median (*horizontal black line*), interquartile range (*box*) and the maximum/minimum values (*tails*)

Both ellipsoid (*R* ^2^ = 0.86, *p* < 0.001) and perimeter (*R* ^2^ = 0.87, *p* < 0.001) estimation of volumes correlated with maximal diameter, following a cubic distribution (Fig. 5b and c). Hardy numbers (*rho* = 0.78, *p* < 0.0001), Hardy letters (*rho* = 0.75, *p* < 0.0001) and the Knosp grades (*rho* = 0.78, *p* < 0.0001) also correlated with tumour volume, although correlations were less strong (Fig. 6).

## Discussion

Presently, maximal tumour dimension (e.g. max diameter) is recommended for use across oncology to assess tumour size and to monitor response to treatment. With advances in technology, tumour volume calculation continues to gather greater focus as it more accurately measures tumour size. It is also recognised that small changes in diameter can grossly underestimate tumour growth. However, the time-consuming calculation required for tumour volume had limited its translation into clinical practice [2, 6].

In this surgical series of pituitary adenomas, both the ellipsoid and perimeter methods of tumour volume estimation closely correlated. Pre-existing measures of pituitary tumours (i.e. the maximal tumour diameter, Hardy and Knosp grades) also correlated with tumour volume, although the relationship was less strong.

In this study, the perimeter method identified slightly larger volumes than the ellipsoid method, consistent with the findings of Sorenson et al. [15]. The discrepancy was larger for the giant tumours, which may assume more complex shapes, given the potential restriction to tumour growth as a result of adjacent bony and neuro-vascular structures surrounding the pituitary fossa. Without direct physical measurement, it is not possible to conclude which method was most accurate; however, logically one would assume that this would be the more comprehensive perimeter method. The slight underestimation of volume by ellipsoid method is not that surprising, although the lower inter-observer error was more favourable than with the perimeter method.

Various automated methods have also been evaluated to estimate tumour volume, but at present are considered less accurate when compared with manual planimetry, although advances continue to be made [1, 6, 16].

The clinical importance of pituitary tumour volume in patient management is not always absolute. As a typically benign tumour, intervention is usually guided by development of clinical symptoms as opposed to tumour volume specifically. Therefore, quantitative methods may have a greater role in those pituitary adenomas under radiological surveillance. The additional information provided by the Hardy and Knosp grades, such as the extent of chiasm compression and cavernous sinus invasion, may be as equally if not more important in influencing the need for intervention and the surgical options.

In the present study, we excluded patients previously operated on. In such cases, the interpretation of the MR images with respect to identifying residual adenoma can be challenging due to the presence of post-surgical changes, leading to greater inter-observer bias. Nevertheless, surveillance of pituitary tumours may benefit from volumetric analysis, since a simple diameter may under-represent a change in tumour volume in response to therapy or watchful waiting [8–10, 12]. Whilst automated methods are limited by relative inaccuracy and availability of software and given the time-consuming nature of manual segmentation, the use of geometric models such as the ellipsoid method, with good accuracy and low inter-observer variability may be clinically more useful for the present [16].

## Conclusions

Pituitary tumour volume can be estimated by a variety of methods. Pituitary tumour volume can be quantitated by the perimeter method or, slightly less accurately, by the ellipsoid method. Qualitative methods such as the Hardy or Knosp grades may provide other clinically important information, such as chiasmal compression or cavernous sinus invasion. In clinical practice, these different methods of assessing pituitary tumour size would likely provide different and potentially equally useful information.
